# Chronic depression: development and evaluation of the luebeck questionnaire for recording preoperational thinking (LQPT)

**DOI:** 10.1186/1471-244X-11-199

**Published:** 2011-12-20

**Authors:** Tanja Kühnen, Franziska Knappke, Tanja Otto, Stephanie Friedrich, Jan P Klein, Kai G Kahl, Michael Hüppe, Valerija Sipos, Ulrich Schweiger

**Affiliations:** 1 Department of Psychiatry and Psychotherapy, University of Luebeck, Ratzeburger Allee 160, 23538 Luebeck, Germany; 2 Department of Anesthesiology, University of Luebeck, Ratzeburger Allee 160, 23538 Luebeck, Germany; 3 Department of Psychiatry, Social Psychiatry and Psychotherapy, Carl-Neuberg-Straße 1, 30625 Hannover, Medical School, Germany

## Abstract

**Background:**

A standardized instrument for recording the specific cognitive psychopathology of chronically depressed patients has not yet been developed. Up until now, preoperational thinking of chronically depressed patients has only been described in case studies, or through the external observations of therapists. The aim of this study was to develop and evaluate a standardized self-assessment instrument for measuring preoperational thinking that sufficiently conforms to the quality criteria for test theory.

**Methods:**

The "Luebeck Questionnaire for Recording Preoperational Thinking (LQPT)" was developed and evaluated using a german sample consisting of 30 episodically depressed, 30 chronically depressed and 30 healthy volunteers. As an initial step the questionnaire was subjected to an item analysis and a final test form was compiled. In a second step, reliability and validity tests were performed.

**Results:**

Overall, the results of this study showed that the LQPT is a useful, reliable and valid instrument. The reliability (split-half reliability 0.885; internal consistency 0.901) and the correlations with other instruments for measuring related constructs (control beliefs, interpersonal problems, stress management) proved to be satisfactory. Chronically depressed patients, episodically depressed patients and healthy volunteers could be distinguished from one another in a statistically significant manner (p < 0.001).

**Conclusion:**

The questionnaire fulfilled the classical test quality criteria. With the LQPT there is an opportunity to test the theory underlying the CBASP model.

## Background

A standardized instrument for recording the specific cognitive psychopathology of chronically depressed patients has not yet been developed. This is a missing link between theory and practice because chronic depressive disorders seem to differ from episodic depressive disorders. Additionally new psychotherapies which are specialized in the treatment of chronically depressed patients have been developed in the past years [1,2]. When examining the effectiveness of therapies having a standardized instrument is an important necessity, since without a standardized specific instrument it cannot be determined whether the specific underlying pathology is modified by the applied therapy. This consideration led to the idea of developing a standardized instrument which contributes on the one hand to diagnosing chronic depressive disorders by identifying the process and quality of preoperational thinking and which on the other hand facilitates therapeutic decisions (in the form of an indication-oriented diagnosis). In addition, the instrument should also serve to evaluate therapeutic success in changing preoperational thinking (e.g. in the form of an evaluative diagnosis).

The development of the instrument for recording the cognitive psychopathology of chronically depressed patients based on the following deliberations:

Approximately 25 to 30 percent of patients suffering from unipolar depression also suffer from chronic depression [3]. Four forms of chronic depression are distinguished in DSM-IV:

1: Dysthymic disorder, 2: Double depression, i.e. single episodes of major depression or recurring major depression and additional dysthymic disorder, 3: recurring major depression that lasts longer than two years without full remission between episodes, and 4: major depression, chronic i.e. major depression that has persisted continuously for a period of more than two years. A fifth profile is presented by double depression/chronic major depression, i.e. where patients fulfill the criteria both for a double depression and a chronic major depression [4].

In the 80's and early 90's it was discussed whether chronic depressive disorders were personality rather than affective disorders. This discussion implies a developmental dimension for chronic depression. Today it is assumed that chronic depressive disorders are a separate category within mood disorders [2]. Common to all five courses is that depressive symptoms last two or more years without any symptom-free periods lasting for more than two months [5].

Chronic depressive and episodic depressive disorders differ in many ways: studies have shown that chronic depressive disorders start at a younger age, chronically depressed patients have a higher comorbidity of axis I/axis II disorders and a more pronounced tendency towards suicidality than episodically depressed patients [2,5,6]. Chronic depressive disorders lead to a more pronounced impairment of psychosocial function [3].

In addition, chronically depressed patients report more negative experiences with significant others than episodically depressed patients [7,8]. Another important distinction is that chronic-depressive disorders are more difficult to treat pharmacologically and psychotherapeutically than episodic depressive disorders [9-13].

The Cognitive Behavioral Analysis System of Psychotherapy (CBASP), developed by James McCullough, is a psychotherapeutic method that is specifically tailored for chronic depressive disorders [1]. One important foundation for CBASP is Piaget's theory of cognitive development. Piaget presented a stage theory for cognitive development from infancy to adolescence [14]. Piaget considered development to be interplay between assimilation and accommodation, the result of which is adaptation. Regarding chronically depressed patients, the second stage of Piaget's cognitive development theory is important. This stage, the so-called preoperational stage, which children experience between the ages of 2 and 6 years, is characterized by the following features: the elementary feelings are spontaneous and the behavior is therefore impulsive. At this stage the child can not yet think logically and there is a focusing on one or a few aspects. The child is egocentric, i.e. he/she is unable to take the perspective of others [14].

McCullough concluded from his observation of chronically depressed patients that such patients are somehow retarded in the preoperational stage. He put forward the following hypothesis: the patients think in a pre-logical manner, i.e. they draw conclusions directly from a prejudice without checking the prejudice itself or any alternative hypotheses. They allow no logical explanations and act entirely in an egocentric manner. As a result of this egocentric world perspective, they express themselves by talking in a monologue manner. This retardation in the preoperational stage becomes a problem if patients are faced with adult tasks: chronic depressive patients do not adequately focus their interpersonal behavior on any anticipated consequences [1].

The reason that chronically depressed patients are arrested in the preoperational phase is considered to be the result of a trauma during childhood or other unfavorable circumstances. This leads to arrested social-interpersonal development. This arrested development is seen particularly in patients with an early-onset of depression. In the case of a late-onset of chronic depression, it is assumed that emotional stress leads to a deterioration of the cognitive-emotional functioning and thus to a reversion to the preoperational stage [1].

The efficacy of CBASP has been systematically investigated:

In the multicenter study (n = 681, 12 treatment centers) of Keller et al. CBASP in combination with psychopharmacotherapy was significantly superior compared to the individual monotherapies (CBASP alone or psychopharmacotherapy alone) [15]. Another multicenter study by Kocsis et al., in which the use of CBASP amongst other alternatives was assessed as an augmentative strategy to psychopharmacology, revealed that CBASP in addition to pharmacotherapy led to response rates of the same order as the comparator conditions (supportive therapy with pharmacotherapy or optimized pharmacotherapy) [16]. Manber et al. showed that the ability to achieve a criteria fulfilling situation analysis was related to the reduction in depressive symptoms during the course of treatment [17].

German studies as well show that CBASP seems promising. In a randomized pilot study Schramm et al. included 30 patients with early-onset chronic depression. The patients were randomized to 22 sessions of CBASP or Interpersonal Psychotherapy (IPT) provided in 16 weeks. While the primary outcome (score on the 24-item Hamilton Rating Scale for Depression (HRSD) assessed post treatment by an independent blinded evaluator) was not significant, secondary measures (remission (HRSD ≤ 8) rates and the Beck Depression Inventory (BDI)) showed relevant benefits of CBASP over IPT [18].

While the efficacy of CBASP is investigated, the assessment of preoperational thinking in adulthood suffers from a lack of appropriate methodology. Up until now, preoperational thinking of chronically depressed patients has only been described in case studies or through the external observations of therapists [19].

Only few studies have tried to test the hypothesis that chronically depressed patients show particular characteristics in cognitive psychopathology. Wilbertz et al. investigated 16 chronically depressed patients with early-onset depression and compared them with 16 healthy controls using a "ToM"-test (the MASC - Movie for the Assessment of Social Cognition), a self-assessment questionnaire for the detection of empathy (the IRI - Interpersonal Reactivity Index) and a structured assessment of preoperational behavior by the therapist. The findings suggested that the chronic depressed patients did not significantly differ from healthy subjects in their ToM-performance. In the estimation of empathy the chronically depressed patients were classified as being inferior to healthy control subjects. In addition, the therapists were able to observe a range of preoperational behaviors amongst the patients [19]. Zobel et al. studied chronically depressed patients (n = 30) using the "cartoon picture story"-test. They were able to show that patients differed significantly in their ToM-performance from healthy subjects. However, after control for logical memory and working memory, ToM-performance was not able to predict patients as such [20].

One criticism of findings from the ToM is that the materials used may not be suitable for investigating ToM-deficits in adulthood and that more appropriate methods still need to be developed [19].

The assessment of preoperational thinking in adulthood also suffers from a lack of adequate tools. There is no instrument directly measuring preoperational thinking in adults.

Thus our study has three aims:

To develop a questionnaire: the Luebeck Questionnaire for Recording Preoperational Thinking (LQPT) for assessing preoperational thinking in adults.

To subject the items of the developed instrument to an item analysis to assess them according to classical test theory criteria and to compile a final test form.

To check the final test form for validity and reliability and to check whether the questionnaire is able to distinguish between episodically and chronically depressed patients.

## Methods

### Construction of the preliminary form of the LQPT

A self-assessment tool was developed since these can usually be used very cost- and time-effectively. The items were constructed on the basis of Situational Judgment Tests (SJT), which are frequently used for screening by human resources departments [21]. Within a SJT, the candidate is confronted with difficult situations he/she might encounter in everyday work. Based on their behavior in these situations it is assumed that candidates will show this behavior in later, similar situations. The situations are described and provided with alternative answers, e.g. with options for deciding how the problem should be reacted to. The candidate is then tasked with classifying the alternative answers according to their effectiveness ("What should you do?"), or to say how they would behave ("What would you do?"). According to McDaniel et al. the SJT are suitable for predicting work performance [22].

The decision to construct our items in a similar way resulted from the following consideration: we wanted to measure preoperational thinking and as such skills mainly in interpersonal situations. For this purpose short stories were constructed with two reaction or thought alternatives, whereby one represented the application of a high level of preoperational thinking and the other represented the application of a low level of preoperational thinking. The answer to be given was the choice that best illustrated how the individuals reacted or thought in the situation (see Additional file 1). These therefore concerned behavior-related, and not knowledge-related, items.

Preoperational thinking illustrated the following characteristics:

*Snapshot perspective *(adherence to the perception of the immediate environment: only sees the current moment, this is seen as a repetition of a negative past and a predictor of the future).

*Prelogical thinking *(a conclusion is reached from a prejudice without any intermediate steps; uninfluenced by the logical reasoning of others).

*Egocentrism *(inability to take the perspective of others and to see one's view as one amongst many).

*Lack of thinking in a perceived functionality manner *(lacking awareness that one's own behavior can entail consequences on one's environment).

*Lack of Empathy *(lack of capacity for empathic communication).

It was assumed that these characteristics all constitute and record a unified concept. The scores of the items are all summed to produce a total value. That means that when the reaction is chosen which is the preoperational one a "0" is given. When the reaction is chosen which is the non-preoperational one a "1" is given. A low total value means a high level of preoperational thinking, while a high total value means that the level of preoperational thinking is low.

An item pool was created which consisted of a total of 22 items. In a first step the created items were classified according to their suitability by the authors and an external expert (JP McCullough). During this process, no items were eliminated, but several changes were made to the wording. In a second step the 22 items were presented to 10 patients to check the extent to which the items could be understood and processed. Here it was found that the instructions could be understood and processed by the depressed patients. As a last step the order of the items was randomized.

### Evaluation of the LQPT

#### Sample

For this study we planned a total of 30 patients with episodic depression, 30 patients with a chronic depressive disorder and 30 healthy volunteers.

We included subjects between 20 and 75 years. All patients with the following forms of chronic depression were included in the group "chronic depression": 1. dysthymic disorder, 2. double depression, 3. recurrent major depression lasting longer than two years without full remission between episodes, 4. major depression, chronic and 5. double depression/chronic major depression. The group of "episodic depression" included patients for whom a major depression or recurrent major depression was present, without the criterion for chronicity having been met. The patients were all in-patients of the Dept. of Psychiatry and Psychotherapy, University of Luebeck, Germany. All of them were included in the study before the start of their therapy. Included in the group of "healthy volunteers" were all subjects in whom there was no clinically significant mental illness. An exclusion criterion for the "healty volunteers" was the existence of any mental illness in the present or the past.

The basis for diagnosis was the "Diagnostic and Statistical Manual of Mental Disorders-DSM-IV [4]. Four trained psychologists conducted the "Structured Clinical Interview according to DSM-IV: axis I and II (SCID)". The diagnosis of the patients was confirmed by board certified psychiatrists.

An official approval was given by the ethic committee of the University of Luebeck, Germany.

#### Item analysis

The items were designed to be of moderate difficulty and selectivity in order to ensure a maximum difference in responses. The item analysis was evaluated for the total sample and separately for the individual groups, since from the manner of the construction of the LQPT it was expected that for healthy subjects, higher indices of difficulty would be achieved than was the case for chronically or episodically depressed patients. It was also expected that the difficulty indices for chronically depressed patients were the lowest of all the groups. The selectivity and difficulty indices were computed with SPSS Version 17.0.

#### Reliability and validity analysis

In a second step, after the selection of the items and collation of the final test form, the *reliability *(internal consistency, split-half reliability) and *validity *(construct validity, discriminative validity) were checked. Since only a single concept was recorded, a high internal consistency was expected.

Correlations were expected with:

Socially determined externality, fatalistic externality and internality measured with the *Competence and control beliefs scale *(FKK): The FKK is a German adaptation of the Levenson's IPC Scale. Here a connection to the lack of thinking in a perceived functionality manner is possible. A low value in the LQPT should be associated with a high externality (FKK-P and FKK-C) and a low internality (FKK-I) [23].

Negative and positive coping skills acquired with the *Stress Coping Questionnaire *(SVF) [24]. Chronically depressed patients generally use self-blame and wishful thinking as the main strategies for coping with stress [1]. A low value in the LQPT should be associated with an increased incidence of negative coping strategies.

Interpersonal problems and dealing with other people acquired by the *Inventory for Interpersonal Problems (IIP-D) *[25]. A connection to the "snapshot perspective" class of characteristics is possible. A low value in the LQPT should be associated with increased interpersonal problems.

Additionally the Beck depression inventory (BDI) was used to record the severity of the depression [26]. The aim was to analyze if the severity of depressive symptoms has a significant impact on performance in the LQPT.

To analyze this it was planned to carry out an ANCOVA. For testing the differences between the three groups it was planned to apply an ANOVA.

## Results

### Sample

The total sample includes 90 subjects, whereby each of the three groups (chronically depressed patients, episodic-depressive patients and healthy subjects) included 30 subjects with 15 men and 15 women. To avoid effects of educational level and cohort effects, the subjects were parallelized in sequence of the highest academic achievement and age. The chronically depressed patients were on average 42 years old (range: 64 years to 22 years; SD: 12.28), episodically depressed patients were on average 43 years (range: 71 years to 22 years; SD: 12.05) while the healthy subjects were on average 38 years old (range: 67 years to 18 years; SD: 11.66).

### Item analysis and item selection

Table 1 illustrates the difficulty indices both for the entire sample and separately for the three groups as well as the selectivity coefficients. Before calculating the discriminatory power, a part-whole correction was performed to prevent an overestimation of the selectivity.

**Table 1:** Difficulty indices (P) and selectivity for the LQPT

Item	Total (N 90)		Chronic (N 30)		Episodic (N 30)		Healthy (N 30)		Selectivity (N 90)
	*P*	*SD*	*p*	*SD*	*P*	*SD*	*P*	*SD*	
1	.86	.354	.73	.450	.90	.305	.93	.254	.341
2	.79	.410	.43	.504	.93	.254	1.00	.000	.656
3	.77	.425	.50	.509	.83	.379	.97	.183	.617
4	.63	.485	.63	.490	.60	.498	.67	.479	.236
5	.81	.394	.60	.498	.90	.305	.93	.254	.470
6	.62	.488	.30	.466	.70	.466	.87	.346	.493
7	.59	.495	.30	.466	.53	.507	.93	.254	.564
8	.80	.402	.60	.498	.87	.346	.93	.254	.390
9	.90	.302	.73	.450	.97	.183	1.00	.000	.560
10	.77	.425	.53	.507	.83	.379	.93	.254	.341
11	.82	.384	.63	.490	.87	.346	.97	.183	.490
12	.68	.470	.40	.498	.63	.490	1.00	.000	.644
13	.90	.302	.70	.466	1.00	.000	1.00	.000	.536
14	.77	.425	.43	.504	.90	.305	.97	.183	.739
15	.83	.375	.63	.490	.87	.346	1.00	.000	.505
16	.80	.402	.63	.490	.77	.430	1.00	.000	.582
17	.90	.302	.77	.430	.93	.254	1.00	.000	.503
18	.79	.410	.63	.490	.77	.430	.97	.183	.581
19	.87	.342	.70	.466	.93	.254	.97	.183	.614
20	.68	.470	.47	.507	.70	.466	.87	.346	.556
21	.78	.418	.67	.479	.70	.466	.97	.183	.472
22	.91	.286	.87	.346	.93	.254	.93	.254	.006

Twenty items revealed a satisfactory selectivity. The item analysis led to the decision that items 4 and 22 (see Additional file 1) would not be included in the final test form. Item 22 showed an excessively high difficulty index, particularly for the chronically depressed patients (0.87) as well as an inadequate selectivity (0.006). Item 4 also showed a low selectivity (0.236), but it also showed an excessively low difficulty index (0.67) for the healthy volunteers.

Borderline suitability was also shown for item 6 (low difficulty index of 0.87 for the healthy subjects) and item 13 (difficulty index of 1.00 in the episodically depressed patients). These two items, however, remained in the final test form since their content was highly representative of the construct.

### Reliability analysis of the final form

The split-half reliability was 0.885. Cronbach's alpha as a measure of internal consistency was 0.901. The inter-item correlation was 0.381. The high internal consistency allows concluding that the test is very homogeneous and that it measures the same characteristic facets.

### Validity analysis of the final form

#### a) Construct validity

The correlations with the IIP, SVF and FKK indicate a number of systematic relationships. Negative correlations for the final form existed for the severity of interpersonal problems (IIP total score, r = -0.761, p < 0.01), fatalistic externality (FKK-C; r = -0.511, p < 0.01) and social externality (FKK-P; r = -0.504, p < 0.01). Positive correlations were shown with positive coping strategies (SVFpos, r = 0.560, p < 0.01) and internality (FKK-I, r = 0.580, p < 0.01).

#### b) Discriminative validity/know-groups validity

The means of the three groups in the LQPT are illustrated in Table 2. The maximum possible score of the final test end was 20.

**Table 2:** Means in the LQPT (final form) differentiated according to group

	mean	minimum	maximum	SD
Healthy	19.20	16.00	20.00	1.19
Episodic	16.53	9.00	20.00	3.51
Chronic	11.40	2.00	20.00	4.83

Statistical tests:

Welch test: 41.667, df1 2, df2 44.517, p < 0.001

A one-way analysis of variance was carried out with SPSS 17.0. Because of unequal variances and the small sample size we used Welch-Test. The analysis showed that the values differed significantly in the three groups at a level of 0.01 (Welch test: 41.667, df1 2, df2 44.517, p < 0.001). Eta^2 ^was 0.466.

Power analysis was made with PASS 2002 (Power Analysis and Sample Size Software for Windows). Power is the probability of rejecting a false null hypothesis. The post-hoc Power analysis revealed that the total sample of 90 achieves 100% Power to detect differences among the means vs. the alternative of equal means using an ANOVA with a 0.01 significance level.

For the Post-Hoc analysis Tamhane's T2 was used. The group of chronically depressed patients differed significantly from the group of episodic depressive patients (p < 0.001) and the group of healthy subjects (p < 0.001). The group of episodic depressive patients differed significantly from the group of healthy subjects (p = 0.001).

The analysis of covariance, in which the severity of depressive symptoms recorded with the Beck Depression Inventory (BDI) was checked, showed that the severity of depressive symptoms had a significant impact on performance in the LQPT (F(1, 86) = 16,506; p < 0.001). Correlations between the BDI ant the LQPT are shown in Table 3. After testing these covariates, the chronically depressed patients still differed significantly (p = 0.002) from the episodic-depressive patients.

**Table 3:** Correlations between BDI and LQPT (final form)

	TOTAL (N 90)	CHRONIC (N 30)	EPISODIC (N 30)	HEALTHY (N 30)
Spearman-Rho (2-tailed)	-.710**	-.456*	-.196	-.232
				

** significant (0.01); * significant (0.05)

## Discussion

The aim of this study was to develop a standardized instrument to measure preoperational thinking that was consistent with test quality criteria. The present study shows that the "Luebeck Questionnaire for Recording Preoperational Thinking (LQPT)" is a valid and reliable instrument. Extensive research performed ahead of this study showed that no instrument exists to date which can measure preoperational thinking in adulthood. The LQPT is therefore the first successful attempt to develop a standardized self-assessment tool for recording preoperational thinking in adulthood.

The LQPT is able to distinguish between chronically and episodically depressed patients at the level of preoperational thinking. As a valuable consequence with the help of the LQPT the special subgroup of chronically depressed patients could be described more thoroughly in future research. As such, the LQPT may in future be a valuable contribution to the study of the effectiveness of therapies. In addition, the LQPT could be used to systematically investigate the specific pathology of chronically depressed patients. Up until now the preoperational behaviors or thoughts of chronically depressed patients have usually only been described in case studies or in the form of external observations [19]. Finally, it would be interesting to find out whether the LQPT even provides the opportunity to predict who will respond or who will not respond to CBAP or other therapies.

Further important steps in the evaluation of the LQPT, which in part arise from the limitations of the present study, include a replication of the results in other patient samples:

Here, only german in-patients were assessed, however, it also needs to be determined whether the results can be replicated for out-patients. Furthermore the total sample of 90 is small to test the psychometric properties of a new questionnaire. Consequently additional studies with larger sample sizes must be done.

It also needs to be checked whether the results are specific to chronically depressed patients, or whether the results can also be seen in patients with a general impairment.

In order to assess validity it would also be useful to check whether there is an association between the LQPT and the ability to carry criteria-consistent situation analyses according to the CBASP concept. Here one should assess whether the LQPT value achieved is related to the extent of preoperational behavior as recorded by external observation. This would help judging the construct validity of the LQPT.

Further studies to assess the reliability of the LQPT shall also be important:

The aim was to record a uniform construct. The internal consistency (0.901) indicates that one characteristic could be reliably illustrated. A factor analysis resulted in no clear and meaningful multifactorial solution, but did show that the test appears to be more heterogeneous than is to be expected from a one-dimensional construct. Further studies must be done to investigate this.

It will also be important to compare the LQPT with "theory of mind"-assessments. The LQPT records e.g. "egocentrism" as an inability to take the perspective of others and react accordingly. This represents a parallel to the ToM-concept. As an adaptive function of the ToM it has often been stated 1) that it serves to predict and explain observable behavior by assessing the so-called "mental states" (desires, intentions, needs) and 2) that the "mental states" are excluded from cues [27]. The results of two studies show that chronically depressed did not significantly differ from healthy subjects in their ToM-performance [19,20]. Perhaps chronically depressed patients presumably have the ability to make assumptions about the internal mental states of others and therefore have no deficits in theory of mind. The problem may be that they are not able to formulate them correctly because they focus only on specific aspects.

Studies intended to take these additional important steps for evaluating the questionnaire are already in planning.

## Conclusions

Overall, the results of this study showed that the LQPT is a useful, reliable and valid instrument to measure McCullough's assumed preoperational thinking in chronically depressed patients. The questionnaire fulfilled the classical test quality criteria and can indeed be used for indicative and evaluative diagnostics in future.

## Competing interests

The authors declare that they have no competing interests.

## Authors' contributions

TK and US have made substantial contributions to conception, design and the first draft of the manuscript. TK, FK, TO and SF have made substantial contributions to acquisition of data. MH, TK and FK have made substantial contribution to the data analysis and interpretation. JPK, VS and KGK have made substantial contribution to the literature analysis. All authors have been revising the manuscript critically for important intellectual content. All authors have given final approval of the version to be published.

## Pre-publication history

The pre-publication history for this paper can be accessed here:

http://www.biomedcentral.com/1471-244X/11/199/prepub

## Supplementary Material

Additional file 1 **Additional file 1 contains the first version of the LQPT and includes the 22 original items**. Click here for file

## References

[B1] McCullough JP Treatment for chronic depression. Cognitive behavioral analysis system of psychotherapy 1994 Guilford Press: New York 10.1002/jclp.10176 12858425

[B2] Klein DN Chronic Depression: Diagnosis and Classification Curr Dir in Psychol Science 2010 19 96 100 10.1177/0963721410366007

[B3] Dunner D Acute and maintenance Treatment of chronic depression J Clin Psychiatry 2001 62 10 16 11310814

[B4] American Psychiatric Association Diagnostic and Statistical Manual of Mental Disorders DSM-IV 1994 American Psychiatric Press: Washington, DC

[B5] Klein DN Shankman SA Rose S Ten-year prospective follow-up study of the naturalistic course of dysthymic disorder and double depression Am J Psychiatry 2006 163 872 880 10.1176/appi.ajp.163.5.872 16648329

[B6] Gilmer WS Trivedi MH Rush AJ Factors associated with chronic depressive episodes: a preliminary report from the STAR-D project Acta Psychiatr Scand 2005 112 425 433 10.1111/j.1600-0447.2005.00633.x 16279871

[B7] Akiskal HS Dysthymic disorder: Psychopathology of proposed chronic depressive subtypes Am J Psychiatry 1983 140 11 20 6336637 10.1176/ajp.140.1.11

[B8] Lizardi H Klein DN Quimette PC Reports of the childhood home environment in early-onset dysthymia and episodic major depression J Abnorm Psychology 1995 104 132 139 10.1037//0021-843x.104.1.132 7897035

[B9] Thase ME Long-Term treatments of recurrent depressive disorders J Clin Psychiatry 1992 53 32 44 1522078

[B10] Thase ME Reynohds CF Frank E Response to cognitive-behavioral therapy in chronic depression J Psychotherapy Pract Res 1994 3 204 215

[B11] Torpey DC Klein DN Chronic depression: update on classification and treatment Curr Psychiatry Rep 2008 10 458 464 10.1007/s11920-008-0074-6 18980728

[B12] Keller MB Gelenberg AJ Hirschfeld RMA The treatment of chronic depression: Part 2. A double-blind randomized trial of sertraline and imipramine J Clin Psychiatry 1998 59 598 607 10.4088/JCP.v59n1107 9862606

[B13] Kocsis JH Pharmacotherapy for chronic depression J Clin Psychol 2003 59 885 892 10.1002/jclp.10180 12858429

[B14] Inhelder B Piaget J The Growth of Logical Thinking from Childhood to Adolescence: An essay on the Construction of Formal Operational Strucutures 1999 Routledge: New edition

[B15] Keller MB McCullough JP Klein DN A comparison of nefazodone, the cognitive behavioral-analysis system of psychotherapy and their combination for the treatment of chronic depression N Engl J Med 2000 342 1462 1470 10.1056/NEJM200005183422001 10816183

[B16] Kocsis JH Gelenberg AJ Rothbaum BO Cognitive Behavioral analysis System of Psychotherapy for Augmentation of Antidepressant Nonresponder in Chronic Depression: The REVAMP Trial Arch Gen Psychiatry 2009 66 1178 1188 10.1001/archgenpsychiatry.2009.144 19884606 PMC3512199

[B17] Manber R Arnow B Blasey C Patient's therapeutic skill acquisition and response to psychotherapy, alone or in combination with medication Psychol Med 2003 33 693 702 10.1017/S0033291703007608 12785471

[B18] Schramm E Zobel I Dykierek P Kech S Brakemeier EL Külz A Berger M Cognitive behavioral analysis system of psychotherapy versus interpersonal psychotherapy for early-onset chronic depression: A randomized pilot study J Affect Disord 2011 129 109 116 10.1016/j.jad.2010.08.003 20822814

[B19] Wilbertz G Brakemeier EL Zobel I Exploring preoperational features in chronic depression J Affect Disord 2010 124 262 269 10.1016/j.jad.2009.11.021 20089311

[B20] Zobel I Werden D Linster H Theory of mind deficits in chronically depressed patients Depress Anxiety 2010 27 821 828 10.1002/da.20713 20577984

[B21] McDaniel MA Hartman NS Whetzel DL Grubb WB Burke MJ Situational judgment tests, response instructions and validity: a meta-analysis Personnel psychology 2007 Blackwell Publishing 63 84

[B22] McDaniel MA Morgeson FP Finnegan EB Use of Situational Judgment Tests to Predict Job Performance: A Clarification of the Literature J Applied Psychology 2001 86 730 740 10.1037/0021-9010.86.4.730 11519656

[B23] Krampen G Verlag für Psychologie Fragebogen zu Kompetenz- und Kontrollüberzeugungen 1991 Dr. C. Hogrefe: Göttingen, Toronto, Zürich

[B24] Janke W Erdmann G Kallus W Verlag für Psychologie Stressverarbeitungsfragebogen (SVF) 2002 Dr. C. Hogrefe: Göttingen, Toronto, Zürich

[B25] Horowitz LM Strauß B Kordy H Verlag für Psychologie Inventar zur Erfassung interpersonaler Probleme (IIP-D) 2000 Dr. C. Hogrefe: Göttingen, Toronto, Zürich 10593141

[B26] Beck AT Ward C Mendelson M Beck Depression Inventory (BDI) Arch Gen Psychiatry 1961 4 561 571 10.1001/archpsyc.1961.01710120031004 13688369

[B27] Bretherton I Frye D, Moore C Intentional communication and the development of an understanding of mind Children's theories of mind 1991 Hillsdale, New Jersey: Erlbaum 49 7322211213

