# Supplementary material for: Chronic depression: development and evaluation of the luebeck questionnaire for recording preoperational thinking (LQPT)
Source: BMC Psychiatry. 2011 Dec 20;11:199. doi: 10.1186/1471-244X-11-199 (PMC3264535; doi:10.1186/1471-244X-11-199)
Supplement: Additional file 1 — contains the first version of the LQPT and includes the 22 original items. [file 1471-244X-11-199-S1.DOC]

**Additional file 1**

**Luebeck questionnaire for recording Preoperational Thinking LQPT (first version)**

**Name: ____________________________ Date: __________________**

**Age: ______________________________ Therapist: ______________**

**Gender: ___________________________**

***________________________________________________________________________***

Instructions: On the following pages you will find descriptions of various situational scenarios or events that involve just you or you and other persons. At the end of each scenario, please “circle” the group of statements (*item set* #1 or #2), or “circle” the statement (statements numbered #1 or #2) that best express how you are most likely to react AT THAT MOMENT. The strong temptation will be to select the *item set* or the *statement* that best expresses how you might like to react. Don’t. Be as honest with yourself as you can throughout the questionnaire when you circle your answers.

**Please answer ALL parts of the questionnaire. Don’t skip any items.**

**_______________________________________________________________________**

*1.) Scenario:* My partner and I agreed to go to my favorite restaurant that night for a candlelight dinner. We haven`t met for a long time, because both of us had have a lot of work to do. Thus I was very happy to meet him that night. After work, my partner calls and tells me that he/she has just had an urgent call from his/her mother about a problem that he/she has to take care of immediately. He/she apologizes and says that he/she will not be able to go out to eat tonight. *(circle the number that best expresses how you feel like reacting right now…)*

1. You’ve just ruined my evening.

vs.

2. I’m sorry about this evening but what is going on with your mother? I want to hear about it.

*2.) Scenario:* Yesterday, I lost my ID card. I looked everywhere and even feared that it had been stolen. I went to bed last night after an exhausting search for the card and woke up this morning feeling and thinking…. *(circle the item set that best expresses how you feel like reacting right now)*

1) These sorts of things always happen to me. I will always be dogged by bad luck.

I don’t know what to do.

Losing the ID card makes me feel like such a failure.

vs.

2) I should go to the police and tell them that my ID card is missing – that will make me feel better.

I will go back over my steps yesterday and see if I dropped it someplace.

Somehow I’ll work this out – I’ll remain confident.

*3.) Scenario:* I am currently out of work, and I have been job hunting. I met a friend in the mall and he told me about his dissatisfaction with his work and that he is having some serious conflicts with several of his colleagues. They are doing some things he does not like. He also said that he is thinking about quitting. He tells me he has been offered another job but the pays is less. *(circle the number that best expresses how you feel like reacting right now)*

1. Nothing ever works out for me. I’ll never find employment anywhere.

vs.

2. My friend is having a difficult time at work. I wonder if he felt my concern for his situation – I hope so.

*4.) Scenario:* I have had a difficult day at work. Several unexpected calls came in from dissatisfied customers. My close friend came to the front door after work and I invited him in. He tells me that he had become very angry with his two children a short while ago and yelled at them. He said he frightened both of the girls. He is very upset about his reaction. *(circle the number that best expresses how you feel like reacting right now)*

1. Happened to me too. Don’t bother about it.

vs.

2. You seem very upset. Tell me what happened and what your children did.

*5.) Scenario:* Earlier today I was downtown shopping and got on a city bus to return home. I was trying to carry too many bags. When I got on the bus, I had to buy my ticket, but I couldn’t find my money. The bus driver became impatient and asked that I hurry up. He told me that he had a time schedule to keep and if I couldn’t find the money I should catch the next bus. *(circle the item set that best expresses how you feel like reacting right now)*

1) I always screw up – I can’t win.

No one is considerate when it comes to me.

I’ll probably never make it back home until very late tonight.

vs.

2) This situation is a mess.

I have to ask the bus driver for more time to find my money.

Maybe I need to get off the bus, find my money, and catch the next bus.

*6.) Scenario:* My girlfriend and I share an apartment. She returned from shopping two days ago and said she wanted to do me a favor and put the food-stuffs in the storeroom. I said, “Fine, but make sure you keep the storeroom neat and tidy when you put up the food”. I like the storeroom neat and didn’t want to have to come in and straighten it up after the food was put away. Today, both of us came home together after going grocery shopping. We were both very tired and wanted to have a restful evening. My girlfriend took the grocery bags and left them sitting unpacked all over the storeroom. She also knocked over several items that I had stored neatly in the corner. *(circle the number that best expresses how you feel like reacting right now)*

1. I have to do everything- no one ever thinks of me and what I want.

vs.

2. I will remind my partner of my request about leaving the store-room neat and tidy. Maybe we will have to renegotiate who does what.

*7.) Scenario:* My neighbor is having his annual birthday party. He has invited me to come to his party every year now for 10 years in a row. This year I was not invited. *(circle the number that best expresses how you feel like reacting right now)*

1. I knew my neighbor never really liked me – this just proves it.

vs.

2. I’m going to call my neighbor; I really want to go to his party.

*8.) Scenario:* I have a meeting with my boss to discuss his six months evaluation of my work. He says that overall he is pleased with my performance. But he has recognized that I have been making mistakes during the past week. He asks me to be more careful in the future. *(circle the number that best expresses how you feel like reacting right now)*

1. My boss will never appreciate what I do on this job.

vs.

2. I have had trouble concentrating lately, and I’ll tell my boss why.

*9.) Scenario:* I’m traveling to visit my friend who recently moved and who is living in another city. I suddenly realize that I forgot to get my map. I had left it on the dining room table. I also realized that I took a wrong turn and now I am lost. *(circle the item set that best expresses how you feel like reacting right now)*

1) I never get anything right – I hate myself.

I’m a loser, and I’ll probably get lost.

I’ll never work this out – I’ll always mess up like this.

vs.

2) I’ll buy another map at the next petrol station.

I can call my friend and ask for directions – don’t give up.

I will be very careful not to do this again – I will learn from this mistake

*10.) Scenario:* I had a very busy day at work, and I have finally come home tired and exhausted. All I want to do is fix some hot tea and rest. After making tea, I pull out a blanket and lie down on the sofa. My girlfriend comes in and asks how I am. I tell her how tired I am – that all I want to do tonight is rest. She begins to suggest activities that the two of us can do for an evening out. *(circle the number that best expresses how you feel like reacting right now)*

1. No one respects my feelings, they just think of themselves.

vs.

2. I’ve got to let my partner know I just want to rest.

*11.) Scenario:* I am having a dinner party and have invited my friends and family members. The morning of the party, one of my friends calls and tells me that he cannot come. He says something “urgent” has come up and he will have to miss the party. He also said that he will call me tomorrow and tell me what is going on. *(circle the item set that best expresses how you feel like reacting right now)*

1) No one likes me.

Others always disappoint me.

I cannot rely on my friends.

vs.

2) I’m sorry my friend will not be here.

I hope my friend is all right.

I will call my friend tomorrow and check on him.

*12.) Scenario:* I am eating lunch with my office colleagues. One man in the group tells us about a pub that he feels we ought to visit. I remain quiet during the discussion of the pub. Several around the table suggest going to the pub that night – I still remain quiet and say nothing. When I get home from work that afternoon, I become irritated and angry because I would have liked to go to the pub with the guys and no one asked me to join in. *(circle the number that best expresses how you feel like reacting right now)*

1. No one likes me – I’m always left out.

vs.

2. I didn’t make what I wanted to do clear to my friends. I’ll just go by the pub.

*13.) Scenario:* I enter a new store to purchase a magazine. After looking around, I cannot find the magazine rack. Next, I find a store clerk and ask where the magazine rack is. The clerk tells me that the magazines are not in his department and that he has no idea where the rack is. He turns around and leaves without trying to assist me further. *(circle the item set that best expresses how you feel like reacting right now)*

1) People are just not helpful. I should never have asked for help.

I’ll always fail with people – no one could ever like me.

vs.

1. This is not a helpful employee. I will look for someone who can help me.

If I can’t find someone to help, I’ll go to another store.

*14.) Scenario:* I have invited a lady I met recently to go to the cinema with me this Friday. I was not sure she would be interested in me or would want to go on a date. I was very happy when she accepted the invitation. On Friday, she called and told me that something had come up and she would have to break the date. *(circle the number that best expresses how you feel like reacting right now)*

1. She would not have liked me anyway.

vs.

2. I am sorry she cannot go. I’ll call her again.

*15.) Scenario:* I am entering a shopping mall shortly before the mall is scheduled to close. Then, I buy several cartons of milk and while I am leaving the store inside the mall, I drop one of the sacks and a carton of milk breaks open and milk spills all over the floor of the store. The store owner sees what happened and becomes angry and says some harsh words to me. He says loudly that I should have been more careful and that now he has to stay after hours and clean up my mess. *(circle the item set that best expresses how you feel like reacting right now)*

1) I never have good luck.

I’m so unskillful.

No one will ever understand me.

vs.

2) This is a very difficult situation for me.

I’ll apologize for the trouble I’ve caused the owner.

I’ll ask the shop owner if I can tip her in compensation for her work.

*16.) Scenario:* My brother is getting married and he asked me to be in his wedding. If I attend the wedding, it will mean taking a two-day leave of absence from work. I had been given several demanding tasks to complete at work and they carried time deadlines that could not be changed. My boss already told me that this work must be completed on time. Completing the work on time will make attending the wedding difficult. *(circle the number that best expresses how you feel like reacting right now)*

1. I’ll never be able to work out leave-time with my boss.

vs.

2. I’ve got to talk to my boss and see if there is anything I can work out.

*17.) Scenario:* My boss gave me a job assignment and as well as a deadline to complete the work. The assignment came at a very busy time and unfortunately, I had several other tasks to complete; therefore, I didn’t finish the assignment on time. I went to my boss and told her that I had not completed the work. She was very understanding. She said to me: “Things like this happen but be sure you finish the work by 9 AM on Monday.” *(circle the item set that best expresses how you feel like reacting right now)*

1) My boss must be really angry with me.

My boss just feels sorry for me – I’m such a loser.

I can’t let myself trust that she’s just being helpful.

vs.

2) My boss really understood my jammed schedule.

I’ve got to get this task finished right away.

My boss’ reaction surely helped me out – I feel relieved.

*18.) Scenario:* My colleague, who is single, and I are in my office discussing the upcoming holiday work schedule. She says she plans to take off work at the exact same time my family and I want to take off for a holiday trip. We argue over the dates and then I tell her that she is being selfish and not thinking of what my family and I want to do. She abruptly leaves my office. *(circle the number that best expresses how you feel like reacting right now)*

1. I can’t work anything out with anyone – I’m just a failure when it comes to people.

vs.

2. I may have offended my colleague by what I said. I want to call her and talk about my reaction before she leaves town.

*19.) Scenario:* I am about to travel internationally and have to obtain a new passport. I went to the passport office and sat down. After waiting 15 minutes to be called, I suddenly realized that I needed to pull a “number” from a dispenser as customers are being called in numbered order. I go up to an empty counter and tell the clerk that I forget to pull a number. I ask if I could be seen without having a number since I had already been waiting for 15 minutes. The clerk tells me in an impolite voice to go back and pull a number and wait my turn. *(circle the item set that best expresses how you feel like reacting right now)*

1) I am so stupid.

Bad luck follows me wherever I go.

No one likes me – everyone gives me a hard time.

vs.

2) I messed up not picking a number.

I will go get a number right now.

I’ll have to go stand in the back of the line.

*20.) Scenario:* I am not feeling well and think I am getting sick. I decide to visit a friend and get the name of a doctor I can see. I also want to get out of the house thinking that I might feel better. I arrive at my friend’s home and he tells me that he cannot talk because he is going to bed and has a very bad headache. He closes the door and I leave. *(circle the number that best expresses how you feel like reacting right now)*

1. Nothing ever works out for me.

vs.

2. I’m sorry that my friend is sick. I’ll talk to him when he’s feeling better. I still need to get the name of his doctor.

*21.) Scenario:* I have just moved to another city in order to begin working at a new job. I’m walking in the front door for the first time. I’ll put my luggage down and look at the flat. I suddenly begin to feel lonely and strange in these new surroundings. *(circle the item set that best expresses how you feel like reacting right now)*

1) I should not have moved.

I always end up having bad luck – it will happen here too.

I’ll never feel at home here.

vs.

2) I will have to work out plans to live in a new place.

I’ll check out my new flat.

It might be difficult for me at first, but I’ll adjust and feel okay about the flat.

*22.) Scenario:* I promised a couple who lives in my apartment building that I would loan them my car for the weekend. My wife and I had planned to stay home all weekend and work in the garden. Unfortunately, I had forgotten that my son had a football match on Saturday night and I had promised him that I would attend his game and drive him and his friends to the game. I told my friends that I cannot loan them the car. What am I likely to say to my friends? *(circle the number that best expresses how you feel like reacting right now)*

1. Sorry, but I cannot loan you my car.

vs.

2. I know this is going to be a problem for you, but I cannot loan you my car. I just found out that my son has a football match that I promised him I would attend.

**Thanks for answering the questions!**
